# A Rare Case of Spontaneous Fungal Peritonitis Caused by Candida lusitaniae in a Patient With Necrotizing Pancreatitis

**DOI:** 10.7759/cureus.40237

**Published:** 2023-06-10

**Authors:** Bianca Varda, Mustafa Alani, Shifat Ahmed

**Affiliations:** 1 Internal Medicine, St. Joseph's Hospital and Medical Center, Phoenix, USA; 2 Internal Medicine, Loyola University Medical Center, Chicago, USA; 3 Gastroenterology, Honor Health, Scottsdale, USA; 4 Gastroenterology, Creighton University School of Medicine, St. Joseph's Hospital and Medical Center, Phoenix, USA

**Keywords:** fungal peritonitis, necrosectomy, gi endoscopy, upper endoscopy, fungal pancreatitis, pancreatitis causes, candida

## Abstract

*Candida lusitaniae* is a rare cause of peritonitis most commonly associated with peritoneal dialysis patients. Pancreatitis is one possible cause of ascites with a low serum ascites albumin gradient. Herein, we present a case of spontaneous fungal peritonitis caused by *Candida lusitaniae* in a patient with necrotizing pancreatitis. The patient was treated with antifungal medication, while her pancreatitis was managed endoscopically with necrosectomy. She improved clinically and was discharged in stable condition.

## Introduction

Fungal peritonitis (FP) is a rare infection with a mortality rate of up to 30%. It is most commonly caused by *Candida *species, specifically *C. albicans *and *C. parapsilosis*. Studies have shown that FP is associated with necrotizing pancreatitis, and patients with more severe pancreatitis based on the Acute Physiology and Chronic Health Evaluation (APACHE II) score are at increased risk of developing FP. Antibiotic prophylaxis or treatment has also been shown to increase the risk of developing FP [1,2]. *Candida lusitaniae* is a fairly rare cause of peritonitis. A PubMed search using the keywords "*Candida *+ *lusitaniae *+ peritonitis" revealed only nine results, most of which were associated with peritoneal dialysis patients [3,4]. Here, we present a case of spontaneous fungal peritonitis caused by *Candida lusitaniae* in a patient with necrotizing pancreatitis.

This case was previously presented as a poster at the American College of Gastroenterology's (ACG) Annual Scientific Meeting in 2021.

## Case presentation

A 33-year-old woman with a history of chronic pancreatitis secondary to alcohol use disorder was transferred to our hospital due to a three-week history of left-sided abdominal pain with radiation to the rest of her abdomen and left flank. During this time, she also reported nausea and bilious emesis.

**Figure 1:**
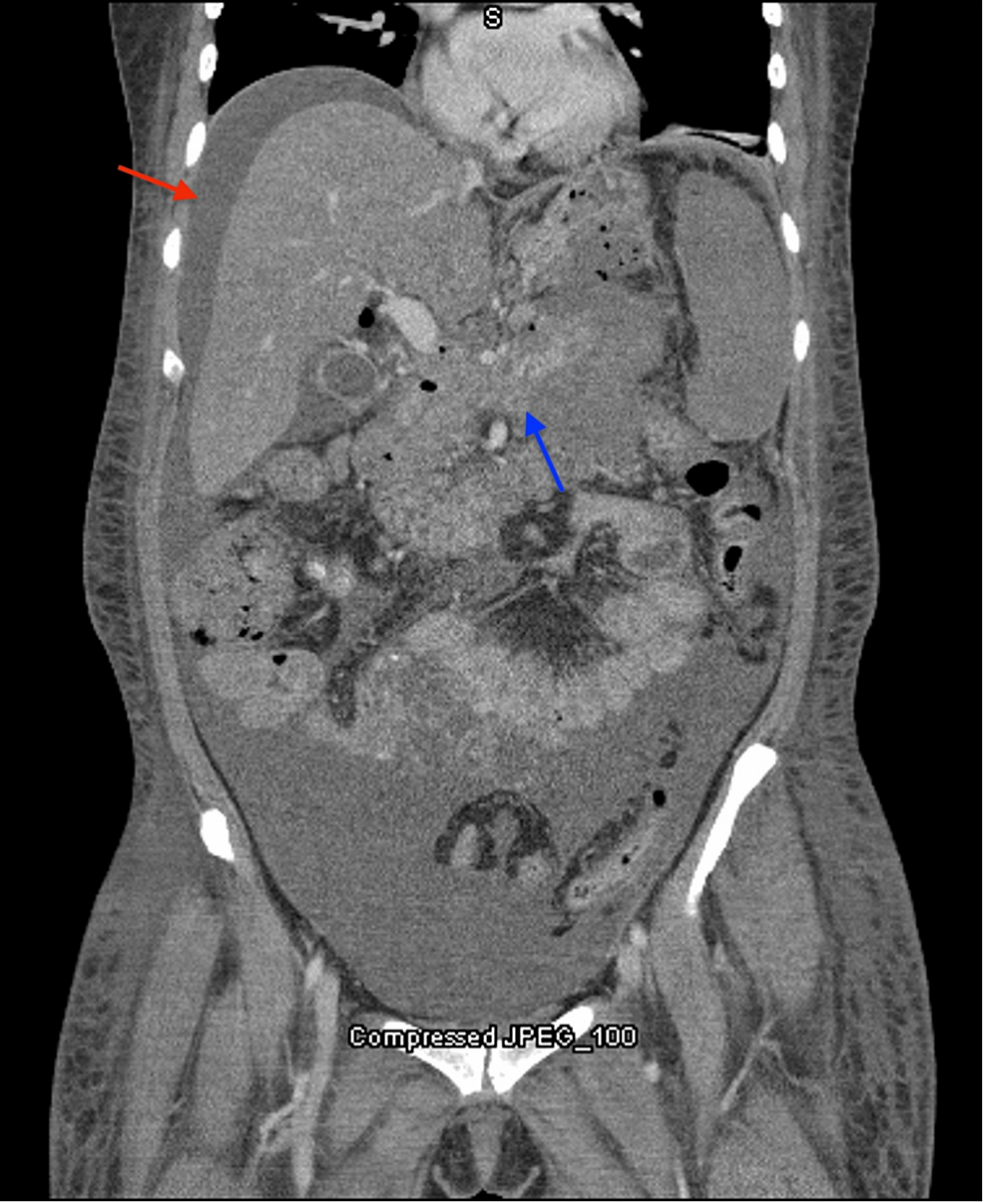
The CT scan shows ascites (red arrow) and the peripancreatic fluid collection (blue arrow).

On presentation, the patient was afebrile and hemodynamically stable. The physical examination was remarkable for a moderately distended abdomen with diffuse tenderness to palpation. Labs were significant for a white blood cell (WBC) count of 54.0 thousand/uL, hemoglobin (Hgb) of 9.9 gm/dL, total bilirubin of 2.2 mg/dL, alanine aminotransferase (ALT) of 66 U/L, aspartate aminotransferase (AST) of 151 U/L, and alkaline phosphatase of 473 U/L. Computed tomography angiography (CTA) was obtained, which showed severe pancreatitis with hemorrhagic peripancreatic fluid and a moderately-sized fluid collection extending along the greater curvature of the stomach with a hypoperfused spleen (Figure 1).

After reviewing images with interventional radiology, it was determined that there was low suspicion for a splenic artery pseudoaneurysm. The patient subsequently underwent esophagogastroduodenoscopy (EGD) with endoscopic ultrasound (EUS) and cystgastrostomy tube placement for further management of pancreatitis with walled-off necrosis. She also required an EGD and necrosectomy two days later. Following the procedure, she continued to complain of abdominal pain and had a persistently elevated white count despite broad-spectrum antibiotics. Her abdomen also remained distended, and she was found to have increasing ascites.

While the ascites was initially presumed to be secondary to pancreatitis, the increased fluid volume and persistent white count prompted further evaluation. Fluid studies obtained during paracentesis revealed a nucleated cell count of 7,906/uL with an 83% polymorphonuclear leukocyte percentage. Serum ascites albumin gradient (SAAG) was less than 1.1, consistent with pancreatitis, and fluid amylase was unremarkable. The patient was continued on empiric antibiotics with a regimen of vancomycin and meropenem.

A culture from the ascitic fluid later grew *Candida lusitaniae* susceptible to fluconazole, and the patient was transitioned to meropenem and fluconazole. She continued to improve while in the hospital, and her white count declined. She was prescribed a 14-day course of fluconazole; the final duration of treatment was to be determined by clinical response. She was scheduled for outpatient follow-up for repeat imaging of necrotizing pancreatitis to plan AXIOS™ stent removal.

## Discussion

Acute necrotizing pancreatitis is a complication of acute pancreatitis that occurs in roughly 20% of patients [3]. It often involves necrosis of the pancreas and peripancreatic tissues. The most common causes include choledocholithiasis and alcohol abuse. Other causes include hyperlipidemia, drugs, and anatomic abnormalities such as pancreas divisum [4]. Clinical presentation includes symptoms of infection such as fever, tachycardia, and leukocytosis in addition to abdominal pain, nausea, and vomiting [4].

Necrotizing pancreatitis is associated with significant morbidity and mortality. Pancreatic inflammation can be associated with pancreatic fluid collections, walled-off necrosis, and pseudocyst development [3]. Initial diagnosis is made with radiographic imaging such as contrast-enhanced computed tomography (CT), which may show fluid collections or areas of low attenuation indicating necrosis [4].

Treatment consists of necrosectomy three to four weeks after the initial insult, which provides time for encapsulation of the collections [3]. Another management option includes other minimally invasive techniques, such as percutaneous catheter drainage. Open necrosectomy has fallen out of favor as it is much more invasive. The management of sterile necrosis is usually conservative. However, despite the absence of infected necrosis, there remains significant mortality in these patients due to early multiple organ failure [4]. Patients with infected necrosis benefit from a necrosectomy performed once the fluid collections have matured.

One possible complication of pancreatitis is peritonitis, most commonly spontaneous bacterial peritonitis (SBP). While our patient did not have a previous diagnosis of cirrhosis, peritonitis can also be seen secondary to pancreatitis due to bacterial overgrowth. Patients with liver cirrhosis are at increased risk of developing SBP due to decreased activity of phagocytic cells in the hepatic system [5]. Other hypotheses for the development of bacterial infections in cirrhotics include bacterial overgrowth and immune system deficiencies.

Spontaneous fungal peritonitis (SFP) is much less common but is another complication of advanced cirrhosis [6]. It is also associated with peritoneal dialysis patients [7]. SFP has a high mortality rate, ranging from 56% to 90%. One study by Hwang et al. demonstrated a 73.3% mortality rate during a one-month period in patients with SFP [5,6].

The most common causative organisms of SFP include *Candida albicans*, *C. tropicalis*, and *C. glabrata*, as well as *Cryptococcus neoformans* [1]. *Candida lusitaniae* is a particularly rare cause of SFP, usually seen in association with peritoneal dialysis (PD). It most commonly affects immunocompromised patients and can cause fungemia, peritonitis, and urinary tract infections, among others. It is uncommon and typically susceptible to most antifungals; however, some strains are resistant to amphotericin B or fluconazole [8]. A PubMed literature review of "*Candida *+ *lusitaniae *+ peritonitis" yielded only nine results, most of which were associated with end-stage liver disease (ESLD) patients on PD.

The treatment of SFP involves a regimen of antibiotics and antifungal agents. At times, patients show improvement with the use of antibiotics alone. In other cases, antifungals such as amphotericin or fluconazole are added to the regimen in an attempt to improve outcomes [1,6]. Treatment is determined on a case-by-case basis, but it is important to diagnose SFP in a timely manner so that medications can be tailored based on a patient’s clinical course.

## Conclusions

SFP is a fairly rare complication associated with liver cirrhosis and ESLD. *Candida lusitaniae*, specifically, has only been reported as a cause of SFP in the setting of peritoneal dialysis. To the best of our knowledge, this is the first case of* C. lusitaniae* SFP in a patient with alcoholic pancreatitis without an underlying diagnosis of ESLD or on peritoneal dialysis. 
